# A public health model for bereavement care: learnings from COVID-19 bereavement experiences in Ireland

**DOI:** 10.3389/fpubh.2025.1611824

**Published:** 2025-12-09

**Authors:** Elizabeth Weathers, Fankun Cao, Helen Coughlan, Avril Easton, Orla Keegan

**Affiliations:** 1 School of Nursing, Midwifery, and Health Sciences, University College Dublin, Dublin, Ireland; 2 St. Vincent's University Hospital, Dublin, Ireland; 3 Irish Hospice Foundation, Dublin, Ireland

**Keywords:** bereavement, grief, end-of-life care, funerals, prolonged grief disorder, brief grief questionnaire, public health model, COVID-19

## Abstract

**Background:**

The COVID-19 pandemic severely disrupted bereavement care, posing risks of long-term physical and mental health consequences. To ensure the lived experience was captured during this challenging time, a national survey was conducted to examine the Irish population’s experiences of dying, death, and bereavement during the pandemic.

**Methods:**

A mixed-methods survey study was conducted between November 2021 and February 2022. Using purposive sampling, a sample of adults completed a 38-question survey. Of the 2,259 participants who completed the survey, 1,223 reported one or more bereavements during the COVID-19 pandemic. This sub-sample of bereaved participants completed the Brief Grief Questionnaire (BGQ). Quantitative data from the sub-sample of 1,223 bereaved individuals were analysed using descriptive and inferential statistics. Qualitative responses were analysed using thematic analysis.

**Results:**

Most participants reported that their bereavement experience was negatively affected by COVID-19, with key themes including loss of connection (including lost opportunities to say goodbye), loss of ritual, and valuing community support. Despite altered funeral structures, many participants found comfort from the efforts made by others to honour the deceased. The BGQ results showed that 59.7% screened negative for Prolonged Grief Disorder (PGD), 26.1% were likely to indicate sub-threshold PGD, and 14.2% were likely to have PGD. Significant associations were found between BGQ scores and several variables including lack of support for bereaved people, relationship to the person that died and being with their loved one at time of death.

**Conclusion:**

The COVID-19 pandemic disrupted traditional end-of-life and mourning practices. The society-wide implementation of public health measures sought to prevent the spread of COVID-19, particularly among our vulnerable populations, but had a deep impact on the dying, death, and bereavement experience in Ireland. This paper gives voice to the bereavement experience of families, friends, and communities and highlights ways to support people who are grieving.

## 1 Introduction

On 30 January 2020, the World Health Organisation (WHO) declared the novel Coronavirus (2019-nCoV) a global public health emergency (1). Two months later, what soon became known as COVID-19 was declared a global pandemic (2). By 2021, COVID-19 had replaced stroke as the second-leading cause of death globally (3). As of 16 March 2025, over 7 million confirmed COVID-19 deaths have been documented worldwide, including approximately 9.8 thousand in Ireland (4).

The declaration of COVID-19 as a global pandemic began an unprecedented global public health crisis, with cascading effects on individuals, communities and societies across the world. One significant impact was the disruption to end-of-life care and bereavement practices (5, 6). In an effort to reduce the transmission of COVID-19, a range of public health interventions were implemented globally (7). These included travel restrictions, social distancing, lockdowns, bans on mass gatherings, and isolation and quarantining protocols. Concurrently, fear and human interest became the framing patterns of COVID-19 media coverage (8) and news outlets, and social media became saturated with daily stories and statistics about COVID-related deaths. While COVID-19 became the focus of attention, restrictive public health measures such as imposed lockdowns, social restrictions and the banning of hospital visits were universal and did not discriminate by cause of death. As such, the impact of public health measures on end-of-life care and on rituals of mourning were not limited to COVID-19 deaths. They affected end-of-life care and mourning rituals for everyone. COVID-19 has also underscored the crucial role played by family and friend caregivers in the continuum of care. In the face of overwhelmed health services and restricted access to formal support systems, there is a need to recognize family and friend caregiving and community-based support as vital components of bereavement care in public health strategies.

Much of the literature on grief and bereavement in the context of COVID-19 has come from Europe, the USA, Canada, Iran, and China (9, 10). The majority of these studies employed quantitative methodologies, while others took the form of review articles or conceptual discussion papers (9, 10). Empirical research typically involved participants who had lost close family members to COVID-19, focusing on their psychological and emotional responses. Common findings across these studies include heightened levels of prolonged grief, anxiety, and depression among the bereaved, largely attributed to pandemic-related restrictions, such as limits on hospital visitations, funerals, and social gatherings, which disrupted traditional mourning practices and support systems (9, 10). Several authors have argued that the pandemic may have lasting repercussions on how societies experience death, dying, and bereavement, due to the scale and abruptness of the losses involved (11–13). However, some scholars have challenged this view, suggesting that while COVID-19 had a significant immediate impact, its long-term consequences on the societal experience of death, dying, and bereavement may be more limited than initially predicted (14, 15).

From an Irish perspective, three studies with a focus on bereavement during COVID-19 have been published to date. All these prior studies were conducted with healthcare professionals and support workers. One study describes the implementation and evaluation of a national bereavement helpline by Irish Hospice Foundation (IHF) in response to the pandemic (16). Results of this study found evidence of high levels of distress among callers to the newly developed bereavement support line. For many callers, their distress was caused by their experiences of hospital visiting restrictions, limitations imposed on rituals of mourning and the absence of meaningful relational connections in the context of these restrictions. The negative impact of disrupted relational connections during COVID-19 was also reported in a study on experiences of midwives providing perinatal bereavement care in one Irish healthcare setting during COVID-19 (17). A further study examined bereavement care provision among people working in hospice, community, and hospital settings across the UK and Ireland (18). The collective experience of participants in that study was of a ‘silent epidemic of grief’, caused by the impact of COVID-19 public health restrictions and the consequent lack of access to meaningful relational bereavement support and services (19).

**Figure 1:**
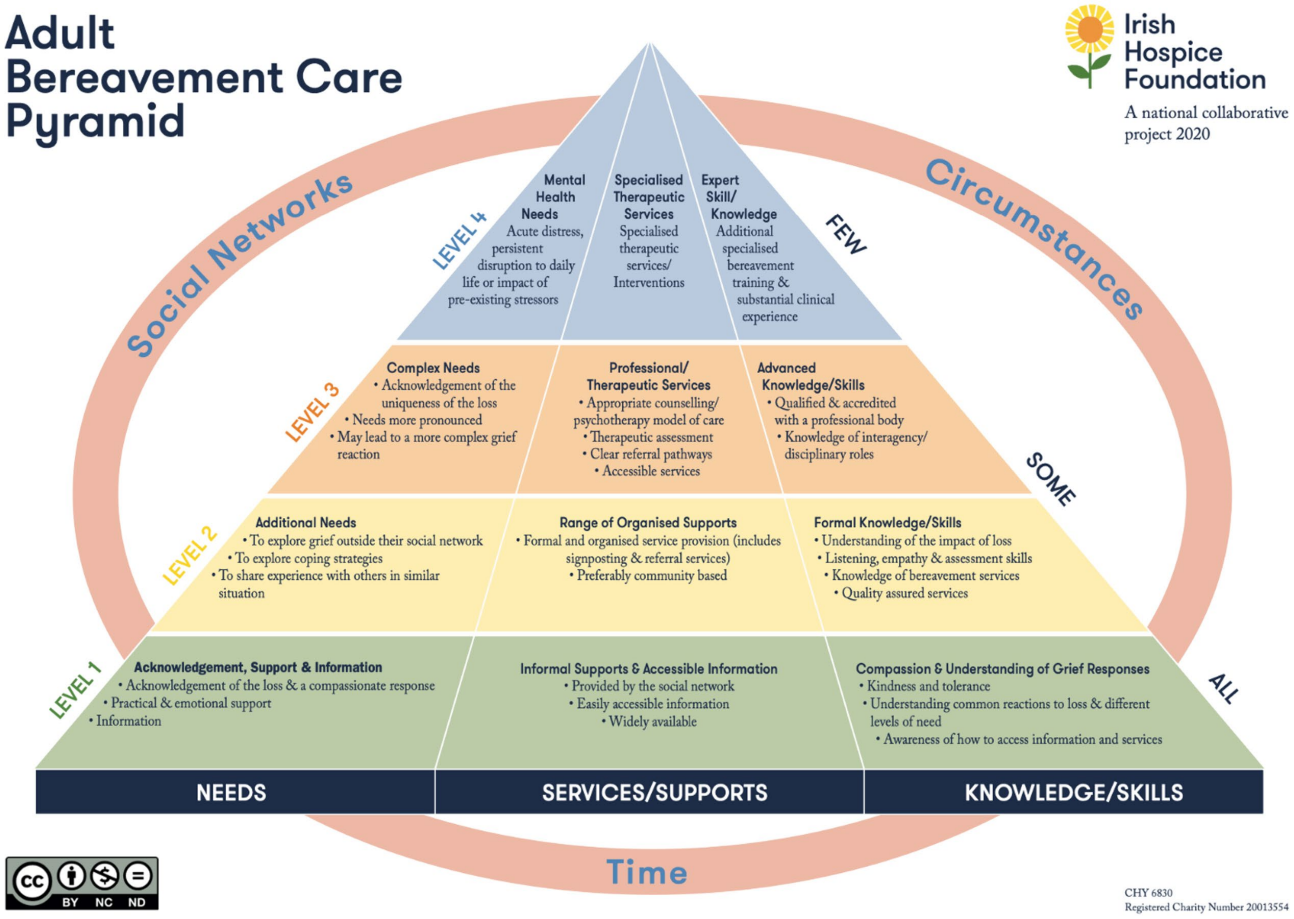
Reprinted with permission from Adult Bereavement Care Pyramid. A National Framework by Irish Hospice Foundation, licensed under CC BY-NC-ND [Source: Adult-Bereavement-Care-Booklet-A-National-Framework.pdf].

Findings from the existing literature capture the multi-level impact of COVID-19 public health restrictions on end-of-life care experiences and on bereaved individuals’ subsequent capacity to access meaningful support (5–7). COVID-19 restrictions disrupted a range of bereavement supports, from informal community-based care to specialist clinical services, reflecting challenges across all levels of support outlined in existing public health bereavement frameworks. One such framework is the Adult Bereavement Care Pyramid (19). Developed by IHF and key bereavement stakeholders, the Adult Bereavement Care Pyramid outlines a structured, evidence-based approach to bereavement care that matches support levels to the specific needs of individuals. It advocates for a stratified approach, where the intensity and type of care are informed by assessed need, to ensure resources are used efficiently and effectively (see Figure 1). This framework shares core principles with the three-tiered public health model for bereavement support developed by Aoun and colleagues (20), which emphasises cost-effectiveness, accessibility, and integrated service delivery. Referencing both models helps to contextualize the varied needs of bereaved individuals during the pandemic and underscore the importance of delivering care that is proportional and responsive to those needs, even during crisis situations.

As a public health bereavement framework, the Adult Bereavement Care Pyramid promotes the importance of balancing the needs of bereaved individuals, including emotional, social, and psychological dimensions, with considerations of cost-effectiveness and accessibility, ensuring that support is proportionate to the level of need and delivered through an integrated framework. During the COVID-19 pandemic, bereavement care services were significantly disrupted, and the individualised support needs of bereaved people were deprioritised in favour of broader public health mitigation efforts, such as infection control and service reallocation (16, 17).

Despite emerging international evidence on the negative impacts of the pandemic on bereavement experiences, no study to date has reported findings from individuals living in Ireland who were bereaved during the pandemic. To address this gap in the literature, the study reported on in this paper presents findings from the only national Irish study undertaken on bereavement experiences during COVID-19. Known as Time to Reflect, the study was commissioned by IHF, a national charity that addresses dying, death and bereavement in Ireland. Framed within the Adult Bereavement Care Pyramid, *Time to Reflect* used a mixed methods approach to collect data from a sample of over 1,200 individuals bereaved during the pandemic. This paper reports on data from the *Time to Reflect* study, i.e., the bereavement experiences of respondents, aiming to quantify the intensity of bereavement reactions experienced by bereaved individuals, exploring how participants described their experiences around the time of death, and to identify factors influencing grief. These findings are then interpreted within a public health framework to consider broader implications for bereavement care and support delivery.

## 2 Materials and methods

*Time to Reflect* was a national survey that aimed to explore the impact of COVID-19 on perceptions and experiences of dying, death and bereavement in Ireland. A key objective of the study was to explore if and how the COVID-19 pandemic affected people’s experiences of dying, death, and bereavement.

### 2.1 Data collection

Data were collected via a survey tool between November 2021 and February 2022. The start date of the survey was 18 months after the declaration of the pandemic and stay-at-home public health mandates in Ireland. It was also one year after the COVID-19 vaccination was made available for vulnerable individuals. Many restrictions remained in place at the time of the survey, including face mask mandates and hospital visiting restrictions. The peak of the death rate in Ireland for COVID-19 was a year prior to the commencement of the survey, making this an appropriate time to capture the bereavement experiences of people living in Ireland during the pandemic. The survey was delivered in both digital (via Survey Monkey) and paper form.

### 2.2 Inclusion/exclusion criteria

Individuals over 18 years old in Ireland during the survey period were recruited by purposive, non-probability-based sampling. The survey was disseminated by IHF through different platforms including IHF events, social media, network connections, and relevant programmes. Paper versions of the survey were also distributed to individuals in nursing home settings. Participants were not required to have experienced a bereavement to participate in the national survey. However, the data reported in this manuscript are specific to those who did experience a bereavement.

### 2.3 Survey instrument

The survey instrument comprised 38 questions across three sections. The first section of the survey Section A examined perspectives on dying, death, and bereavement since the pandemic began. Participants who indicated they were bereaved during this time proceeded to Section B, which focused on understanding participants’ personal experience before and after the death of their loved one. The final section of the survey (Section C) collected demographic and wellbeing information from bereaved and non-bereaved participants. The survey instrument is available to view on the IHF website.^1^

Survey responses were predominately presented in the form of Likert scales. Open-ended questions were also asked throughout the survey. This was to ensure that participants had the opportunity to give voice to their subjective experiences that could not be collected through quantitative questions. Bereaved participants, which are the focus of the current paper, were also invited to complete the Brief Grief Questionnaire (BGQ). The BGQ is a 5-item questionnaire developed to screen for symptoms of Prolonged Grief Disorder (PGD) (21, 22), reflecting intense feelings of grief that are long-lasting and interfere with everyday life. It is a screening instrument and not a diagnostic tool. Individuals scoring between 0 and 4 on the BGQ are considered to screen negative for PGD. Scores between 5 and 7 indicate an individual is likely to report sub-threshold symptoms of PGD, and scores 8 or higher indicate the individual is more likely to meet criteria for PGD.

### 2.4 Data analysis

Quantitative data were analysed using IBM SPSS 27. Descriptive and inferential statistics were used in the analysis. Responses to the five BGQ questions were computed into total scores ranging from 0 to 10. BGQ total scores were grouped into three categories for statistical analysis; negative screen for PGD (0–4), positive screen for sub-threshold symptoms of PGD (5–7), and positive screen for likely PGD (8–10). Relationships between BGQ categories and items reflecting participants’ experiences of the death of a relative were explored in the analysis. Chi-square tests were used to examine associations between categorical variables, and independent samples t-tests were employed for comparisons of continuous variables across BGQ categories. The level of significance was set at *p* < 0.05, with Bonferroni correction applied to control for Type I error across multiple comparisons. These categorical cutoffs are meaningful as they reflect established thresholds used to screen for / identify concern around prolonged grief.

Qualitative data derived from the open-ended survey questions were analysed thematically (23). Two researchers independently coded the data. An inductive coding approach was used where the development of themes and sub-themes were derived from interpreting the data and not based on any pre-conceived ideas of what patterns the researchers would like to see in the data. To ensure that qualitative data was analysed successfully, the six phases of thematic analysis as illustrated by Braun and Clarke were used (23). Firstly, the researchers familiarised themselves with the data by reading and documenting initial ideas (phase 1). The relevant text was then imported into NVivo 12 where the researcher assigned multiple codes using an open and axial coding approach (phase 2). Succeeding this phase, codes were refined and grouped into potential themes along with relevant data/excerpts (phase 3). An in-depth discussion then took place with the extended research team to further review the themes and sub-themes (phase 4), before reaching a collective agreement on the identification and definition of themes and sub-themes (phase 5). Discrepancies in coding were resolved through iterative discussion and consensus, ensuring that interpretations remained grounded in the data and that differing perspectives enriched the analysis. Finally, chosen themes and sub-themes were selected for report inclusion coupled with compelling and relevant excerpts from the open-ended questions.

### 2.5 Ethical considerations

Participants were provided with an information leaflet prior to completing the survey and contact details of the study team were also provided if participants had any questions or queries about the study. Participation in the study was voluntary, and participants could opt out if they wished to. Ethical approval to carry out the study was granted by the Royal College of Surgeons Ireland (REC approval no: 202105022). IHF acted as the data controller when managing the data and was responsible for the protection, storing, and analysis of the survey. The anonymity of participants was prioritised when storing and analysing survey data.

### 2.6 Reflexivity statement

As researchers working in the deeply emotive field of death, dying, and bereavement, and during an unprecedented global pandemic, we recognised the importance of reflexivity in acknowledging our own subjectivity and emotional response. Engaging with hundreds of personal, and often heart-breaking, narratives of loss was challenging at times. The intensity and poignancy of participants’ accounts evoked empathy, sorrow, and a sense of shared vulnerability. We were not outside observers but situated actors, whose own experiences and bereavements during COVID-19 inevitably shaped how data were interpreted and presented. Personal experiences of bereavement among the research team brought added depth and sensitivity to the research process. These experiences strengthened our commitment to honouring the stories and insights shared by participants. In the context of the emotional depth of the work, regular team debriefings were incorporated, to reflect on the data and analysis, while remaining sensitive to the ethical dimensions of working with such material. This reflexive approach deepened our understanding of the data and helped us remain grounded in the human significance of the stories entrusted to us.

We also recognise that our personal and professional proximity to the topic may have introduced certain interpretive biases. For example, a heightened sensitivity to grief-related distress or an inclination to privilege certain narratives over others. To mitigate this, we engaged in ongoing critical reflection as a team and the diverse experience and background of the research team (nursing, social work, psychology, social policy) ensured varied perspectives during analysis. By making this process visible, we aim to honour the trust placed in us by participants, and to contribute to a more transparent, ethically aware, and emotionally attuned bereavement research practice.

## 3 Results

A total of 2,259 adults took part in the *Time to Reflect* survey. Of those, a nested sub-sample of 1,223 participants reported experiencing one or more bereavements during the pandemic. This paper presents both quantitative and qualitative findings from the sub-sample of bereaved individuals only (*N* = 1,223). Findings related to bereavement experiences will be presented with a focus on the following themes: bereaved participants’ experience of dying and death, funerals and mourning rituals, role of bereavement support and community involvement and screening for indicators of PGD.

### 3.1 Quantitative findings

#### 3.1.1 Demographics

Among the 1,223 bereaved participants, 1,078 (88.1%) completed the demographic section of the survey (see Table 1). Of these 1,078, a majority were female (*N* = 753, 69.9%) and aged between 45 and 54 years old (*N* = 322, 29.9%). A majority (*N* = 1,008, 93.5%) described their ethnicity as White Irish. Participants were distributed across rural, urban and suburban settings and almost half of bereaved participants were either married or in a civil partnership (*N* = 514, 47.8%). Participants who did not complete the demographic section (*N* = 145, 11.9%) were retained in the analysis of all other sections of the survey, provided they had responded to those relevant items.

**Table 1:** Demographic variables for bereaved participants.

Variable	*N*	%
Age		
18–24*	73	6.8%
25–34*	115	10.7%
35–44	178	16.5%
45–54*	322	29.9%
55–64*	209	19.4%
65–74*	135	12.5%
75+	27	2.5%
Unknown*	19	1.8%
Total	1,078	100%
Ethnicity		
White Irish	1,008	93.5%
Irish Traveler	1	0.1%
Black Irish	1	0.1%
Any other white background	45	4.2%
Any other black background	1	0.1%
Asian Irish	4	0.4%
Any other Asian background	9	0.8%
Other	9	0.8%
Total	1,078	100%
Gender		
Male	318	29.5%
Female	753	69.9%
Other/non-binary	5	0.5%
Prefer not to say	2	0.2%
Total	1,078	100%
Relationship status		
Single, never married	265	24.6%
Civil partnership or married	514	47.8%
Cohabiting with a significant other	167	15.5%
Separated or divorced	62	5.8%
Widowed	68	6.3%
Total	1,076	100%
Geographic location		
Urban	243	22.5%
Suburban	259	24.0%
Town	227	21.1%
Rural	349	32.4%
Total	1,078	100%

#### 3.1.2 Impact of COVID-19 on the experience of dying

Over 86% of participants (*N* = 976, 86.3%) reported that their experience of a loved one’s death was negatively affected by COVID-19 (see Table 2). Less than 20% of bereaved participants (*N* = 223, 19.6%) were able to spend time with their loved one before they died and over half of the participants (*N* = 717, 63.7%) were not present with their loved one when they died. Most participants reported receiving the support they needed from the professionals involved in the end-of-life care of their loved one (*N* = 273, 24.2%). Nonetheless, this meant that over one in five participants reported not receiving the support they needed from professionals involved in the end-of-life care of their loved one (*N* = 246, 21.8%) and a further 19.2% (*N* = 217) reported that they somewhat or partly received the support they needed from professionals involved in the end-of-life care.

**Table 2:** Experiences before the death of a loved one.

		Responses *N* (%)				
	Total *N*	Yes	Somewhat or partly	No	Do not know	Does not apply
My experience around the time of their death was negatively affected by COVID-19	1,130	718 (63.5%)	258 (22.8%)	115 (10.2%)	14 (1.2%)	25 (2.2%)
I was able to spend the time I wanted with them before they died	1,135	223 (19.6%)	189 (16.7%)	641 (56.5%)	2 (0.2%)	80 (7%)
I was with them at time they died	1,126	267 (23.7%)	34 (3.0%)	717 (63.7%)	5 (0.4%)	103 (9.1%)
The professionals involved in the end of their life gave me the support I needed	1,128	273 (24.2%)	217 (19.2%)	246 (21.8%)	29 (2.6%)	363 (32.2%)

#### 3.1.3 Funerals and mourning rituals

While most respondents were able to attend the funeral of their loved one, almost one in four were not (*N* = 262, 23.5%). Almost 70% of participants (*N* = 765, 68.6%) reported that family and friends were excluded from funerals due to COVID-19 restrictions (see Table 3). Almost half of participants stated they were unable to have the funeral or ritual they would have liked for their loved one (*N* = 531, 47.5%).

**Table 3:** Impact of COVID-19 on funerals and mourning rituals.

		Responses *N* (%)			
	*N*	Yes	Somewhat or partly	No	Does not apply
I was unable to attend their funeral in person because of the COVID-19 restrictions	1,116	262 (23.5%)	68 (6.1%)	697 (62.5%)	89 (8%)
Some family and/or close friends were excluded from the funeral because of the COVID-19 restrictions	1,115	765 (68.6%)	147 (13.2%)	158 (14.2%)	45 (4.0%)
We were able to have the funeral service or ritual we wanted for them	1,119	215 (19.2%)	342 (30.6%)	531 (47.5%)	31 (2.8%)
People in the community still found meaningful ways to honour them on the day of their funeral	1,121	601 (53.6%)	353 (31.5%)	131 (11.7%)	36 (3.2%)
Even with restrictions, some aspects of the funeral were a comfort to me	1,114	608 (54.6%)	310 (27.8%)	145 (13.0%)	51 (4.6%)

Just over half of participants (*N* = 608, 54.6%) reported that, even with the restrictions, some aspects of available funeral rituals were a source of comfort for them. Over half of participants stated that people in their community had found other ways to honour the person who had died (*N* = 601, 53.6%).

#### 3.1.4 The role of bereavement support and community involvement

Over 60% of participants (*N* = 691, 63.6%) reported that the pandemic made their grieving process more difficult, with almost 40% (*N* = 396, 36.7%) reporting that they did not get the support they needed after the death of a loved one because of the public health measures. Over half of respondents (*N* = 580, 53.6%) reported that, despite the public health measures, their family and friends found meaningful ways to support them in their grief (see Table 4).

**Table 4:** Experiences after the death of a loved one.

		Responses *N* (%)				
	*N*	Strongly agree	Agree	Neither agree nor disagree	Disagree	Strongly disagree
The Impact of COVID-19 made my grief more difficult	1,086	280 (25.8%)	411 (37.8%)	253 (23.3%)	83 (7.6%)	59 (5.4%)
Even with restrictions, my family and friends have found meaningful ways to support me in my grief	1,082	79 (7.3%)	501 (46.3%)	325 (30%)	127 (11.7%)	50 (4.6%)
I did not get the support I needed after the death because of the COVID-19 restrictions	1,080	128 (11.9%)	268 (24.8%)	389 (36.0%)	220 (20.4%)	75 (6.9%)

**Figure 2:**
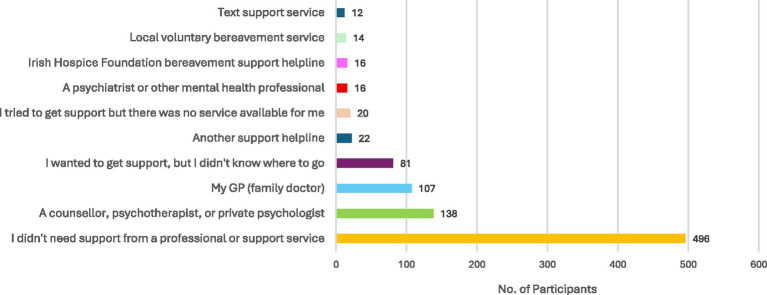
Bereavement support services accessed during the COVID-19 pandemic.

Over half of bereaved participants (*N* = 496, 53.8%) reported that they did not require extra support from a professional or support service (see Figure 2). For those who did require support services, most sought help from a counsellor, psychotherapist, or private psychologist (*N* = 138, 15.0%) or from a GP (*N* = 100, 10.8%). A minority of participants (*N* = 80, 8.7%) reported that they wanted to get support but did not know where to go.

### 3.2 Indicators for possible prolonged grief disorder (PGD) and sub-threshold grief

The mean score among participants who completed the BGQ (*N* = 1,095) was 3.95 (SD = 2.86) (see Table 5). Almost 60% of participants (*N* = 653, 59.7%) scored between 0 and 4 on the BGQ, indicating a negative screen for PGD. Over one in four (*N* = 286, 26.1%) scored between 5 and 7, likely to indicate sub-threshold symptoms of PGD. A further 14.2% of participants (*N* = 156) scored between 8 and 10, indicating that these individuals were more likely to meet criteria for PGD.

**Table 5:** BGQ scores for bereaved participants.

Variable	Bereaved *N* = 1,095	
	*n*	%
BGQ category		
No PGD indicated (0–4)	653	59.6
Sub-threshold PGD indicated (5–7)	286	26.1
PGD indicated (8–10)	156	14.2

#### 3.2.1 BGQ and place of death

No statistically significant differences in BGQ categories were found based on the place of death of the participant’s loved one (see Table 6).

**Table 6:** BGQ and place of death.

Place of death	BGQ category							
	No PGD indicated (0–4)		Sub-threshold PGD (5–7)		PGD indicated (8–10)			
	*N*	%	*N*	%	*N*	%	X^2^ (16)	*p*
Own home (*N* = 207)	140	67.6%	45	21.7%	22	10.6%	18.88	0.275
Hospital (*N* = 245)	139	56.7%	75	30.6%	31	12.6%		
Nursing home (*N* = 121)	82	67.8%	30	24.8%	9	7.4%		
Hospice (*N* = 82)	47	57.3%	19	23.2%	16	19.5%		
Other (*N* = 32)	20	62.5%	9	28.1%	3	9.3%		

*p*-value computed based on chi-square test and level of significance was set to 0.05.

#### 3.2.2 BGQ and relationship to and time spent with the deceased

A statistically significant association was found between BGQ categories and participants’ relationship to the deceased (see Table 7). Indicators of risk of PGD were more prevalent in participants who lost a parent, a spouse, in-law, or a child compared with those who lost other family members.

**Table 7:** BGQ and relationship to deceased.

Relationship	BGQ category							
	No PGD indicated *N* = 639		Sub-threshold PGD indicated *N* = 279		PGD indicated *N* = 152			
	*N*	%	*N*	%	*N*	%	X^2^ (22)	*p*
Parent/Stepparent (*N* = 323)*	165*	25.8%	85	30.5%	73*	48.0%	137.59	<0.001
Grandparent (*N* = 88)	56	8.8%	21	7.5%	11	7.2%		
Sibling (*N* = 67)	32	5.0%	21	7.5%	14	9.2%		
Client/resident/patient (*N* = 35)	25	3.9%	9	3.2%	1	0.7%		
Friend (*N* = 145)*	86	13.5%	49*	17.6%	10*	6.6%		
Other (*N* = 20)	15	2.3%	3	1.1%	2	1.3%		
Extended family member (*N* = 177)*	130*	20.3%	38*	13.6%	9*	5.9%		
In-law (*N* = 95)*	65*	10.2%	24	8.6%	6*	3.9%		
Son or daughter (*N* = 14)*	4*	0.6%	4	1.4%	6*	3.9%		
Spouse/partner (*N* = 39)*	6*	0.9%	14*	5.0%	19*	12.5%		
Neighbour (*N* = 35)	26	4.1%	8	2.9%	1	0.7%		
Colleague (*N* = 32)*	29*	4.5%	3*	1.1%	0	0.0%		
Total	639	100.0%	279	100.0%	152	100.0%		

*p*-value computed based on chi-square test; * indicates significant differences; Bonferroni correction applied to reduce Type I error.

No statistically significant associations were found in BGQ categories between those who reported having the time they wanted with their loved one before their death and those who did not (see Table 8). Conversely, significant associations were found between those who were not (*p* < 0.001). Specifically, those participants who were not with the person who died (10.4%) scored lower on the BGQ than those that were with the person who died (25.8%) (see Table 9).

**Table 8:** BGQ and time spent with the deceased before they died.

I was able to spend the time they I wanted with the person before they died	BGQ category							
	No PGD indicated (0–4)		Sub-threshold indicated PGD (5–7)		PGD indicated (8–10)			
	*N*	%	*N*	%	*N*	%	X^2^ (6)	*p*
Yes	123	55.9%	58	26.4%	39	17.7%	8.31	0.216
No	362	59.2%	172	28.2%	77	12.6%		
Somewhat or partly	99	53.7%	45	25.0%	36	20.0%		
Do not know	1	50.0%	1	50.0%	0	0.0%		

*p-*value computed based on chi-square test; Bonferroni correction applied to reduce Type I error.

**Table 9:** BGQ and being with the deceased at the time they died.

I was with them at the time they died	BGQ category							
	No PGD indicated (0–4)		Sub-threshold indicated PGD (5–7)		PGD indicated (8–10)			
	*N*	%	*N*	%	*N*	%	X^2^ (6)	*p*
Yes	118*	44.7%*	78	29.5%	68*	25.8%*	47.48	<0.001
No	432*	62.7%*	185	26.9%	72*	10.4%*		
Somewhat or partly	20	58.8%	6	17.60%	8	23.5%		
Do not know	2	50.0%	2	50.0%	0	0.0%		

*p*-value computed based on chi-square test; * indicates significant differences between yes and no column values.

#### 3.2.3 BGQ and access to support during COVID-19 pandemic

A statistically significant association was found between BGQ categories and participants’ access to support during the pandemic (see Table 10). Bereaved participants were asked to rate their level of agreement with the following statement: *‘I did not get the support I needed because of COVID-19 restrictions’*. A higher percentage of those who agreed or strongly agreed with the statement were more likely to report indications of PGD and sub-threshold PGD.

**Table 10:** BGQ and access to support during COVID-19 pandemic.

Response to: “I did not get the support I needed because of COVID-19 restrictions”	BGQ category						
	No PGD indicated (0–4)		Sub-threshold PGD (5–7)		PGD indicated (8–10)		
	*n*	%	*n*	%	*n*	%	*p*
Strongly agree (*N* = 128)	34	26.6%	41	32.0%	53	41.4%	< 0.05
Agree (*N* = 268)	121	45.1%	103	38.4%	44	16.4%	
Disagree (*N* = 220)	171	77.7%	38	17.3%	11	5.0%	
Strongly disagree (*N* = 75)	52	69.3%	14	18.7%	9	12.0%	

*p*-value computed using independent samples t-tests and level of significance was set to 0.05.

#### 3.2.4 BGQ and experience of death

Chi-square tests were conducted to determine if associations were found between respondents reporting that COVID-19 prevented the person from having the death they would have wished for and their BGQ category (see Table 11). Respondents that felt COVID-19 prevented the person from having the death they would have wished for had significantly higher indications of sub-threshold PGD relative to those who did not feel that way.

**Table 11:** BGQ and COVID-19 preventing the death the respondent would have wished for the deceased.

COVID-19 prevented them from having the death I wished for them	BGQ category							
	No PGD indicated (0–4)		Sub-threshold indicated PGD (5–7)		PGD indicated (8–10)			
	*N*	%	*N*	%	*N*	%	X^2^ (6)	*p*
Yes	244*	50.8%*	155*	32.3%*	81	16.9%	32.67	<0.001
No	166*	66.7%*	45*	18.1%*	38	15.3%		
Somewhat or partly	123	66.1%	44	23.7%	19	10.2%		
Do not know	33	75.0%	9	20.5%	2	4.5%		

*p*-value computed based on chi-square test; * indicates significant differences between Yes and No column values.

#### 3.2.5 BGQ and ability to have a funeral or ritual

Table 12 shows the chi-square results comparing the ability to have the desired funeral or ritual for the death of the loved one and BGQ category. There were no associations between those who did and those who did not have the desired funeral and BGQ category (*p* = 0.491).

**Table 12:** BGQ and ability to have funeral or ritual the respondent wanted.

We were able to have the kind of funeral service/ritual we wanted for them	BGQ category							
	No PGD indicated (0–4)		Sub-threshold indicated PGD (5–7)		PGD indicated (8–10)			
	*N*	%	*N*	%	*N*	%	X^2^ (6)	*p*
Yes	126	60.9%	52	25.1%	29	14.0%	3.41	0.491
No	293	56.3%	147	28.3%	80	15.4%		
Somewhat or partly	209	62.4%	82	24.5%	44	13.1%		
Do not know	0	0.0%	0	0.0%	0	0.0%		

*p*-value computed based on chi-square test.

### 3.3 Qualitative findings

Open-text responses from participants gave voice to the lived experiences of the sample. Irreparable loss was the superordinate theme identified through thematic analysis, with participants denied the relational connections they needed and valued in the context of dying, death and bereavement. Nested within this main theme were three sub-themes of loss of connection; loss of ritual; and valuing community support. Each of these themes are presented below.

#### 3.3.1 Loss of connection

Loss of connection was evident in the qualitative accounts of participants. Participants described multiple and layered losses to engage in the end-of-life care and bereavement experience. Sub-themes related to this loss of connection are presented below.

##### 3.3.1.1 Lost opportunities to say goodbye

A theme of loss related to the experience of being unable to say goodbye to loved ones was evident across qualitative accounts. COVID-19 restrictions resulted in people being unable to be with loved ones in the final weeks, days, hours and moments of life. Participants shared stories of loved ones and patients dying alone due to capped visiting numbers in hospital and nursing home settings.

*“My brother-in-law died suddenly and unexpectedly from a late diagnosis of cancer. His wife and his children could not sit with him or comfort him but could only look through the glass. Only in his last minutes when he was already in a coma where they allowed at his bedside. They never really got to say goodbye, even worse, they never got to comfort him, tell him they loved him, while he could still hear them. None of us got to say goodbye. There has to be a better way.”*

Another participant highlighted how strictly hospital staff adhered to the restrictions and denied family members any opportunity to visit their loved ones:

*“I lost my mum to COVID-19, 2 years previously (April 2020). She went into hospital with an infection. We were restricted from visiting her for 3 months. Around every corner, every effort was made to keep us out of the hospital.”*

The implementation of restricted visits in hospital settings was undoubtedly difficult for many people. One participant expressed being unable to be with her grandmother when she died and the effect that this had:

*“My Nan got a stroke and was in hospital, she got 3 more strokes afterwards. She was 10 days in hospital altogether before she passed away. I had stressed to the hospital staff that I lived nearly 2 hours from the hospital and if anything happened to her to please ring me. They rang and said she had a turn, I said I’d leave, and they told me not to. By the time I got to the hospital my beautiful grandmother was gone at 94. She had died on her own. I will never forgive myself.”*

##### 3.3.1.2 Alone in grief

Many participants described grieving alone, without access to their informal family, friendship and community supports. Participants reported feelings of loneliness, powerlessness and lack of relational contact as they navigated the period of time after a death:

*“The distance between people really highlighted to me how isolating it can be. I live alone and I struggle with my own emotions. I missed the support of family and friends. I missed the comfort of a hug or a chat over a shared cup of tea.”*

Participants expressed a lack of bereavement support during the pandemic. This included lack of support offered by professionals or generalized difficulties accessing support while grieving. For instance, one participant recalled their experience of visiting their GP to get support following a bereavement and explained how limited emotional support was given:

*“Even if a death occurred prior to the pandemic, for the most part the COVID has had a knock-on effect to those who were grieving when it started. When I went to see my GP as I thought I needed help, he said "of course you don't feel right, you lost both your parents. Keep losing weight" – nothing was offered to help i.e., counselling etc.”*

Another participant shared the experience of paying a counsellor for support during the pandemic, but the service provided did not meet her expectations:

*“I lost a family member who I was very close to in recent times and cannot cope with the loss. I have searched for proper grief counselling without success. All help lines are only open for a few hours during the day whereas those of us who live alone find the evening and night-time the worst to deal with. There is no proper grief counselling for people like me. I paid a so-called counsellor €140 and all he told me was “you are a strong woman you can work it out for yourself”. I am disgusted with the lack of grief counselling particularly for the older generation.”*

Another participant explained how the nurses were very caring towards her mother in hospital but mentioned the disappointment of no support being offered to herself or her siblings when it was needed:

*“At no time were myself or my siblings offered any numbers for support afterwards etc. when we could have done with it. I cannot fault the care the nurses gave to my mom. They were super. Just an observation on the afterwards care for ourselves and the follow up.”*

##### 3.3.1.3 Difficulty supporting others

Participants frequently referred to the difficult experience of being unable to extend their support to family and friends during the pandemic due to preventive measures in place and fear of contracting or passing on COVID-19. For example, one participant recalled the following experience:

*“A friend's Dad passed at the beginning of the pandemic, and I found it hard to know what to do e.g., could I go to the house or funeral? In the end I went to the house but felt awkward because I couldn’t shake hands, hug, or even stand next to the coffin to pay respects.”*

Another participant who was a healthcare worker described the heartbreak of not being able to comfort her family at a funeral during the pandemic in fear of placing her family members at risk of COVID-19.

*“I’ll never forget watching my 86-year-old aunt sob silently and without being able to physically comfort her for fear I’d place her at risk as I’m a nurse working in a hospital with COVID patients.”*

The impact of restrictions on people’s capacity to be with and support loved ones was captired by one participant who emphasised the difficulty of being unable to comfort his wife at a funeral due to the restrictions placed on funeral arrangements during the pandemic:

*“When my wife’s grandmother died, funeral attendance was capped at 10 so I had to sit in the car and watch the funeral via livestream, so I was unable to hold her hand and give her comfort during the mass and this was very difficult.”*

#### 3.3.2 Loss of ritual

The theme of loss was also reflected in the people’s experiences of restrictions to rituals of mourning. Many participants mentioned difficult experiences due to the restrictions that were placed on funerals. The capped attendance numbers, social distancing, and overall new arrangements of funerals made the bereavement experience even more difficult for family members, leaving them in a state of shock:

*“She was lifted by the funeral director and buried two hours later. We left clothes but it is my belief that they did not dress her. They didn’t tell us, and we didn’t ask. I believe that she was placed in a bag, and it was put into a coffin. We walked to the church; kind neighbours lined the route but only the immediate family were allowed into the graveyard. The gate was closed. We were not permitted to carry the coffin. The priest quickly said a few prayers. It was over in a matter of minutes. Almost no one spoke to us as we walked home in shock.”*

One participant reported the difficulty in choosing who could attend funerals and indicated how the funeral did not meet their expectations:

*“It was like a lottery trying to decide who would and wouldn’t attend. We were able to have time with our loved one in a funeral home; however, only a very limited family were able to come and say goodbye. I found this very difficult as it is certainly not the type of funeral we would have intended.”*

Another participant highlighted the grief and pain that is still present due to the restricted funeral arrangements during the pandemic:

*“We never left her side throughout her 9-month battle but were robbed of those last precious times. We got no funeral. 10 of us. We couldn’t carry her coffin or bring up gifts. We had to drive our own cars and pick coffins over WhatsApp messages. I look back over it and it was so surreal. We weren’t surrounded by family and friends and now 18 months later I feel my mom is just forgotten by everyone and they have moved on and I think we just feel stuck in that grief and devastation.”*

Participants emphasised that disrupted or absent rituals of mourning negatively affected their grieving process.

*“When you bury a loved one during a pandemic, like I did… and you turn away from their grave and go home to an empty house, no memorial gathering, no extended family and friends to share the loss or share a memory. It’s by far the loneliest of times. Something as a nation we wouldn’t be used too.”*

Another participant compared the death of her mother before the pandemic with her father’s death during the pandemic and highlighted how the removal of traditional grieving rituals disrupted and impacted the grieving process:

*“It didn’t change my views so much on dying and death, but I probably didn’t understand the importance of ‘rituals’ in the aftermath of the death of a loved one until they were taken away. My mother’s death from cancer almost 5 years ago was a drawn-out lingering process but we had time to process it every step of the way. COVID robbed us of that when it came to our father’s death.”*

Another participant also emphasised how the removal of traditional mourning rituals or norms could have long-term detrimental effects for many people:

*“During the pandemic as we couldn’t attend funerals it was extremely lonely for families to go home to their respective households alone without this step of the grieving process. I believe that grief is delayed in these circumstances and that as a country we will have a lot of people grieving once we get through this time and employers, friends etc. may not be mindful that people need time. This could potentially cause or aggravate some form of depression.”*

#### 3.3.3 Importance of community support

Alongside people’s experiences of loss and disconnection during the pandemic, data from this study revealed the importance that people place on relational connection and community support at times of death and loss. The way in which support was exchanged differed immensely to pre-pandemic times and participants emphasised the value of extending traditional support to grieving individuals:

*“I think the restrictions make it difficult for someone living after a bereavement – the same support networks can’t be there in person. I think that makes the aftermath more difficult – having people around for a while afterwards can help processing, and even help with practical things afterwards – people could do with support then too as the emotions are likely to arise fresh again e.g., dealing with business issues, wills etc.”*

Similarly, another participant spoke of not being able to show support due to restrictions on household visits and funeral attendance:

*“A relative died during the pandemic but not from COVID-19. The family did not have the benefit of the community being able to attend the mass and could not call to houses. Then I realised how important all that is when there is a death.”*

The pandemic reinforced the importance of community support and participants reported how they came to realise the importance of community support:

*“The need to be able to share a death with your community was something I took for granted. Having sadly been at a number of close relatives’ funerals, I see that the funerals with restricted numbers do not permit a family to hear lovely stories about the deceased or feel the support of the community.”*

The value of connection and togetherness as a vital part of the grieving process was highlighted by another participant:

*“I didn’t realize how important it is to have the support of relatives/community when a person dies, but when that is diminished, it can be a difficult process. Irish people have historically been good at grieving and coming together at a difficult time, and when we could not do that, it made it harder.”*

## 4 Discussion

This paper presents compelling findings on experiences of dying, death and bereavement in Ireland during the COVID-19 pandemic. Combining both quantitative and qualitative data from a large survey study in Ireland, findings reveal how relational connections with family, friends and community were restricted, disrupted and, at times severed, at a time when people needed them most. Qualitative findings confirm that the loss of meaningful connection and support was a key source of subjective distress for bereaved persons.

### 4.1 Deaths during COVID-19 and impact on bereaved people

In line with other published studies, findings from this study demonstrate that some participants who were bereaved during COVID-19 experienced significant distress around the time of their relative’s or friend’s deaths (24). Many were unable to spend time with the person in the time before they died and only one in five were with the person when they died. Quantitative findings were supported by analysis of qualitative data from the study, providing a window into the meaning of these experiences for participants and their families. Participants spoke of a sense of abandonment, inconsistent with the priorities of caring and family roles before the pandemic. Participants discussed how these experiences gave rise to feelings of guilt, remorse, and moral suffering, which were also captured in the literature (14, 24).

The visiting restrictions in place across the country caused a major disruption in the normal functioning of hospitals and nursing homes during the pandemic. Qualitative findings emphasise the struggle of bereaved participants, who were often denied the opportunity to be present at their loved one’s bedside in the last moments of their lives. Being present for a loved one’s death is usually desired by family members and is considered an opportunity to say goodbye (25, 26). An interesting finding from this study, was that being present at the time of death was associated with prolonged grief. The reasons for this association are unclear from the data. However, it is possible that the psychological burden of witnessing the death, especially under traumatic and emotionally dysregulated conditions, contributed to dissociative coping responses, which can complicate the grieving process (27). As Diamond argues, trauma that is not mentally processed but instead dissociated can manifest later as unresolved or “repressed” psychological material, intensifying feelings of grief and loss. In such cases, being physically present does not necessarily equate to psychological preparedness or emotional closure (27). Neither the specific location (hospital versus home) nor the cause of death was associated with poorer bereavement outcomes. This suggests that how the death was managed and supported may have played a more significant role. Given that this study was conducted during an unprecedented public health crisis, which severely impacted healthcare staffing and procedures, other contextual factors may have contributed to the experience of prolonged grief. Further exploration of the lived experiences and surrounding circumstances that may have shaped the grief process in these individuals is warranted. This could include further exploration of the overlap between those who reported their relative died at home and those who were present at the time of death.

Two in five (40%) bereaved people felt they did not get the support they needed at their loved one’s time of death. It is possible that protective clothing, limited opportunity for touch, the experiences of uncertainty and communication around what to expect played a role. Also given that the closeness of relationship is associated with PGD it is possible that there is a confounding effect at play. A recent palliative care study also noted the anomalous finding where being present at the time of death was associated with increased use of bereavement support (28). Their findings were not COVID-related and their conclusion about supporting family members to navigate this important time links to the implications of our study.

### 4.2 The role of funeral and universal support in mourning amid the impact of COVID-19

Findings of a rapid review conducted early in the pandemic showed that research into the functions, processes and impacts of funerals for bereaved people is lacking (29). The importance of funerals as shared, collective and honouring rituals was highlighted through the data presented here. For the majority of respondents, the person who died did not have the funeral they would have wished for, and equally respondents were unable to provide the funeral they would have wanted. There is a poignancy in some of the qualitative comments, which illustrate respondents coming to a realisation of the importance of ritual – something that had not been articulated or understood by them before. This rupturing of cultural scripts (thinking of the funeral as a script) can be viewed from two perspectives, i.e., the destabilising loneliness, and isolation experienced is one perspective however these experiences of limited funerals were not associated with higher grief intensity or risk as measured by the BGQ.

A second perspective is one of adaptation and cultural flexibility (30). Even with restricted attendance most people say they found comfort in funerals. Testament to this flexibility is that the majority of bereaved people note that their community were able to find other ways of honouring the deceased. ‘Honouring’ in this way is a vital support function of the funeral, and one that plays a vital role in bereavement care at the universal, level 1 of the public health approach to bereavement care. Community bereavement support or ‘universal support’ extends beyond physical presence to encompass emotional, psychological, and social dimensions. Over half of the respondents said that family and friends were able to meaningfully support them in their grief, even with restrictions. Evidence shows that social support acts as a protective factor, and meaningful connections within a community can significantly mitigate grief-related distress (28). Similarly, informal social support networks play a vital role in maintaining psychological well-being among those who have lost loved ones due to sudden or violent deaths (31). Natural bereavement support fosters sustained emotional and social engagement (32) and key actions of community members active listening, validation, and sharing experiences need to be privileged.

An important feature of universal support was evident in the qualitative data of this work – it was notable that respondents spontaneously reported the difficulty in supporting others as a disturbing experience. Bowe et al. reported on the mental health benefits of community helping during the COVID-19 crisis showing that voluntary caring has benefits for the giver as well as the person supported. While informal bereavement support does not come under this coordinated volunteer support banner, the role of a caring informal supporter, and the subjective impact of being limited in the ability to provide this support should not be underestimated and merits further exploration (33).

### 4.3 Impact of COVID-19 on bereavement support

The survey found that the majority of participants did not seek formal support from a professional or a support service, which is consistent with prior literature that highlights that informal networks of support are more commonly used and sought by the bereaved (20, 34). However, it is also clear from the literature that those who do seek out bereavement support are in fact those most in need (34). A rapid systematic review to identify effective system-level responses to mass bereavement events, including natural disasters, and pandemics, noted the importance of proactively identifying those in need of support, to ensure need is met (35). Almost half of bereavement service worker participants in an Irish study reported an increase in demand for bereavement support services and many reported more complex needs among service users during COVID-19 (36).

### 4.4 Impact of COVID-19 on likelihood of PGD and sub-threshold PGD

Findings indicated that 14% of participants screened positive for likely PGD, which is less than prior studies conducted during the pandemic. Indications of 30 to 87% were identified in a recent systematic review (6). While indications of PGD at 14% are lower than proportions reported elsewhere, the relatively large number of individuals with a positive screen for sub-threshold symptoms on the BGQ show that one in four bereaved people may need closer monitoring. This study used the five item Brief Screening Questionnaire (21) in order to minimize participant burden. It is important to note that the BGQ is a screening tool rather than a diagnostic instrument, and none of the 28 studies of PGD during COVID-19 identified by systematic review use the BGQ and therefore there may be a question on the degree of comparability of rates (6).

Many of the higher PGD rates reported in the literature (6) derive from studies conducted in countries with different healthcare systems, cultural mourning practices, and degrees of service disruption during the pandemic. In contrast, Ireland’s national response—particularly the role of organisations such as the Irish Hospice Foundation in supporting grief literacy, provision of information to professionals working those who were bereaved and advocating for revised consideration of the public health measures —may have helped buffer some of the adverse impacts of pandemic-related loss. Additionally, cultural norms in Ireland around community-based mourning rituals, spiritual care, and familial cohesion may have played a protective role, even when traditional practices were disrupted.

With respect to risk factors, this study confirmed that closeness of relationship was associated with more intense grief (37). As noted, and discussed above, higher indications of PGD were evident for those present at the time of death and for those who self-reported that they did not get the support they needed because of COVID-19 restrictions (41% of those above thresholds for possible or likely PGD did not get adequate support). There are a number of possible reasons for this finding that may be of interest in further studies. For example, even if a relative was present, for periods of time during the pandemic, loved ones were forced to wear full personal protective equipment in hospitals and unable to hold the hand of a loved one as they died. For others, their care of their loved ones at home happened at a time when restrictions meant they did not have access to the typical levels of palliative care support.

BGQ was chosen as a short, validated screening tool for PGD and the findings presented here should be viewed in that context; diagnosis of PGD rates would require further investigation, including use of validated assessment tools and clinical interview reflecting the multidimensionality of PGD (21, 22). Nevertheless, BGQ as a screening tool has merits; a PGD screening study of 1,015 bereaved patients in a primary care setting (38) concluded that the BGQ performed as well as the Inventory of Complicated Grief and was easier and shorter to administer. Currently in Ireland, despite the recognition of the public health approach to bereavement care, no established screening or clinical pathways for referrals to grief treatment exist. The current study has demonstrated the short five questions are acceptable to an Irish grieving population and some consideration could therefore be given to piloting a screening programme in Ireland.

### 4.5 Public health framework and planning for bereavement care

Conceptualizing grief as a public health issue is increasingly recommended in order to prevent health impacts and to apportion the most effective supports for those most in need (20, 34, 39, 40). The model promotes communities being grief literate and supportive to all those grieving as a fundamental basis for healthy grief. This extends to the support provided in the lead up to a death, and at the time of death. The need for a public health approach to bereavement was noted as necessary prior to the onset of COVID-19 but was amplified by a time when the focus on dying, death and grief had never been greater.

IHF has long advocated for a strategic and coordinated approach to bereavement care. Developed by IHF, the Adult Bereavement Care Pyramid provides a national framework for adult bereavement care in Ireland (19). The pyramid suggests that every person who experiences bereavement will have some level of need. Support at all levels of the continuum of grief must be available so that people can access the support when and where they need it. Others will require more intensive support, such as counselling and a few will require support from a specialist therapeutic service.

This structure corresponds closely with the three-tiered model, which divides bereaved individuals into low-risk, moderate-risk, and high-risk categories (39, 41). In both frameworks, the majority of bereaved individuals require only informal or community-based support, while a smaller proportion benefits from structured interventions, and a minority requires specialist care. Aoun’s empirical work provides robust validation for the tiered approach to bereavement care as set out in public health models, including the Adult Bereavement Care Pyramid used in Ireland (20, 34).

A pre-COVID 2015 study demonstrates that 58% of bereaved individuals have Level 1 (low level) needs, 35% were at Level 2 (moderate risk), and 6.4% were Level 3, high-risk or demonstrating criterion scores on the Inventory of Complicated Grief (34).

People who are at risk of complications in their grieving need to be identified and matched to the appropriate supports, while those experiencing difficulties should be able to access appropriate professional help.

The data presented here used the five item BGQ to screen for potential indicators of PGD and to approximate the levels of bereavement support need among those who self-reported a bereavement during the COVID-19 pandemic in Ireland. Most (59.6%) of the bereaved survey respondents were categorised as low risk (BGQ score 0–4) with respect to their BGQ, almost identical to Aoun’s results (34). Using the screening tool, around one in four (26%) were categorised as likely to have symptoms of sub-threshold PGD and 14% were indicated as possible symptoms of PGD, or as definitely requiring further diagnostic investigation for PGD.

The implications of these results are a mixed picture; a positive was that natural support seemed to remain a protective factor for the majority of bereaved through the pandemic, despite the changes and limited interactions while public health measures were in place. However, a sizeable proportion, approximately one in ten people (14%), presented with possible indications of PGD, higher than Aoun’s 6.5% but lower than levels of pandemic bereavements reported in the research literature (24). A further 26% of the Irish sample fit into a moderate risk category and these people require further attention. In summary, approximately 40% of those surveyed were identified by the BGQ as potentially benefiting from additional bereavement support. We use the term “support need” here rather than “bereavement risk” to align with the BGQ’s function as a screening tool rather than a predictive risk assessment or diagnostic tool.

The findings of this report are beneficial for public health practitioners, healthcare workers, family carers, policymakers, and the public. The literature review for this survey report highlighted that there is concern globally regarding the detrimental impact of COVID-19 on the grieving process and the provision of bereavement support, particularly considering the public health measures and restrictions placed on society during the pandemic. Several authors have indicated that there is likely to be long-standing consequences on dying, death, and bereavement due to the profound loss of human life, and that the increase in pathological grief will become a worldwide public health concern, calling it a silent epidemic of grief (14, 42). Others have suggested that the impact may not be as extensive as originally anticipated (29). There is consensus regarding the need for continued research on the topic and close monitoring of people who have been bereaved to identify those most at-risk of developing PGD or other complications, and to intervene early.

Drawing on our findings, which highlighted how restrictions placed on families being with their loved ones, as they approached end of life, was a key source of distress for bereaved individuals, we recommend that future healthcare responses prioritise relational and compassionate care even during public health crises. To mitigate the traumatic experience of bereaved individuals, healthcare systems should facilitate supportive communication that meets the needs of both the dying person and their family, flexible and safe visitation policies wherever possible, and ensure opportunities for loved ones to say goodbye.

Lichtenthal’s concept of transitional bereavement care is a guide for how health and social/community contexts need to link (40). The transitional bereavement care model bridges the gap between healthcare institutions and community-based support systems (40). This model emphasises the importance of continuity of care, ensuring that bereaved individuals receive support as they transition from institutional care to community resources (40). Key components include preventive bereavement care, ownership of bereavement services by institutions, allocation of resources for bereavement support, upskilling of support providers, and implementation of evidence-based practices (40). By integrating these elements, the model aims to address the multifaceted needs of the bereaved, promoting a comprehensive and compassionate approach to bereavement care (40). By integrating these elements, many of which respond directly to the gaps in care and support identified by participants in this study, the model promotes a comprehensive and compassionate approach to bereavement care that addresses both pandemic-specific and broader systemic challenges.

Screening and early intervention for PGD or complicated grief should be incorporated into health policy and practices. Recommendations from the literature include the need to find innovative ways for healthcare workers to connect with and support bereaved persons during a public health crisis (43) and the need to ensure continuity of spiritual and religious activities, as well as social support for patients and their families during a public health crisis.

To address the limited access to bereavement support, health services must be equipped with additional resources for patients and families, and healthcare workers should receive training in social–emotional skills to better support grieving families, and bereavement support groups (both online and in-person) should be expanded. Learning from the pandemic demands that, in normal times as well as in crisis, we identify factors that promote healthy grieving, intervene with those at risk of complications and treat people who are intensely suffering from prolonged or other disturbed grieving processes. It is also important to acknowledge the broader ethical complexity of conducting bereavement research during a pandemic, where public health measures aimed at controlling a highly contagious infectious disease may conflict with individual and relational values around end-of-life care, mourning, and grief. This ethical tension, between protecting the collective good and respecting personal and familial needs, underscores the need for careful ethical reflection on the design and implementation of studies during a public health crises.

### 4.6 Limitations

The survey was cross-sectional in nature and collected data among participants at one point in time. A cohort study that follows participants over a period of time may yield different findings because views and perceptions may change. In particular, longitudinal follow-up, such as repeating the questionnaire one and two years later, could provide valuable insights into the evolving nature of grief and the longer-term impact of bereavement experiences. This would allow for a deeper understanding of trajectories of adjustment and potential delayed grief responses.

The study had limited representation of younger age groups, male participants, non-Irish ethnic background, and foreign nationals residing in Ireland. However, this is aligned with other similar studies. Future research with diverse populations is warranted. Another limitation of the study was the sensitive nature of the survey questions relating to dying, death, and bereavement. This may have impacted on the missing data throughout the survey responses. While we did collect data on several causes of death, including confirmed and suspected COVID-19 infection, cancer, cardiac conditions, and others; this information was not verified via medical records or death certificates. Future research would benefit from incorporating verified clinical data to explore these patterns further. Nonetheless, this survey study is the only nationwide survey conducted on this topic in Ireland.

While the BGQ was selected as a brief, validated screening tool for PGD, several limitations should be acknowledged. The BGQ is a screening rather than diagnostic instrument and does not capture the full multidimensionality of PGD as defined in recent diagnostic frameworks. Its reliance on self-report introduces the potential for response bias and limits the capacity to distinguish between normative grief reactions and clinically significant PGD. Additionally, although the BGQ has demonstrated good sensitivity and specificity in previous studies, further validation within diverse cultural contexts would strengthen its applicability. Consequently, the findings presented should be interpreted as indicative of probable PGD risk rather than confirmed diagnoses, and future research should incorporate comprehensive diagnostic assessments and longitudinal follow-up.

### 4.7 Future research

There is a consensus in the literature that further research on the long-term impacts of COVID-19 is warranted and this research should include large population-based samples, as well as studies that focus on specialised groups. Further research monitoring the severity of grief amongst different groups (e.g., healthcare professionals) would also be beneficial to inform public health responses and practices to support those at risk of developing PGD or complicated grief.

## 5 Conclusion

The COVID-19 pandemic profoundly altered experiences of dying, death, and bereavement, leaving a lasting impact on individuals and society. Public health measures imposed to control the spread of the virus often prevented families from being present with dying loved ones and disrupted traditional mourning rituals, leading to widespread emotional distress. The *Time to Reflect* study is the only Irish investigation on bereavement experiences in Ireland, providing comprehensive insights into the impact of COVID-19 on end-of-life care, grief, and bereavement support. By capturing data from a broad demographic, it offers a robust evidence base to inform bereavement care policies and interventions.

Findings from this study highlight the significant impact of public health measures on funerals and mourning rituals, and characterize the varied experiences of grief and responses to bereavement among individuals in Ireland during the COVID-19 pandemic. A public health approach to bereavement care involves identifying the diverse needs of the bereaved and mapping out a spectrum of responses, including informal and community-based support and organised and professional interventions. In this survey, it was recognised that all bereaved people need compassion, clear information and the support of those around them. However, being cognisant of the difficult factors affecting people who were bereaved during COVID-19, it was important to delve deeper and try to identify what was the possible scale of additional need for support.

The pandemic heightened public awareness of grief and loss and reinforced the importance of dignified end-of-life care. There is an ongoing global concern about the long-term effects of disrupted grieving processes, highlighting the need for sustained research, early intervention, and enhanced bereavement support within healthcare systems. Future policies must ensure that, even in public health crises, human dignity and the need for connection at the end of life remain a priority. Significantly, the findings highlight the indispensable role of family and friend caregivers, who not only provide practical assistance and emotional comfort but also serve as vital connectors within communities, ensuring that the bereaved are not isolated in their grief.

By capturing data from a broad demographic, this study offers a robust evidence base to inform bereavement care policies and interventions. The study’s national scope ensures that findings reflect diverse experiences across different communities, systemic challenges, and the varying support needs of bereaved individuals. Furthermore, as the first large-scale survey of COVID-19-related bereavement in Ireland, it establishes a critical foundation for future research, enabling comparisons over time, which can support the development of evidence-based bereavement care frameworks. Its findings are particularly valuable for public health planning, healthcare service improvement, and policy development, ensuring that bereavement support in Ireland is both inclusive and responsive to the needs of those affected by loss.

## Data Availability

The datasets presented in this article are not readily available because in accordance with the ethical approval granted for this study, all study records including study data will be destroyed in 2026 and therefore, will be unavailable to share. Requests to access the datasets should be directed to OK, orla.keegan@hospicefoundation.ie.

## Footnotes

^1^ https://hospicefoundation.ie/wp-content/uploads/2021/11/IHF_Time-to-Reflect-Survey.pdf
